# The Fungal Pathogen *Candida albicans* Promotes Bladder Colonization of Group B *Streptococcus*

**DOI:** 10.3389/fcimb.2019.00437

**Published:** 2020-01-10

**Authors:** Samuel R. Shing, Anissa R. Ramos, Kathryn A. Patras, Angelica M. Riestra, Sinead McCabe, Victor Nizet, Alison Coady

**Affiliations:** ^1^Collaborative to Halt Antibiotic-Resistant Microbes, Department of Pediatrics, University of California, San Diego, La Jolla, CA, United States; ^2^Skaggs School of Pharmacy and Pharmaceutical Sciences, University of California, San Diego, La Jolla, CA, United States

**Keywords:** *Streptococcus agalactiae*, *Candida albicans*, urinary tract infection, polymicrobial infection, fungal-bacterial interaction

## Abstract

Group B *Streptococcus* (GBS) is a common cause of bacterial urinary tract infections (UTI) in susceptible populations, including pregnant women and the elderly. However, the factors that govern GBS persistence and disease severity in this niche are not fully understood. Here, we report that the presence of the fungus *Candida albicans*, a common urogenital colonizer, can promote GBS UTI. Co-inoculation of GBS with *C. albicans* increased bacterial adherence to bladder epithelium and promoted GBS colonization *in vivo* in a *C. albicans* adhesin-dependent manner. This study demonstrates that fungal colonization of the urogenital tract may be an important determinant of bacterial pathogenesis during UTI.

## Introduction

*Streptococcus agalactiae* (group B *Streptococcus*, GBS) is a common inhabitant of the intestinal and vaginal tract in ~18% of the healthy population and can become pathogenic and cause serious disease in neonates and susceptible adults (Meyn et al., 2009). While the implementation of antenatal screening and treatment of maternal GBS colonization prior to birth has significantly reduced the incidence of early-onset neonatal disease, GBS infection in adult populations remains a persistent problem, particularly with respect to urinary tract infection (UTI). Although the presence of GBS in the urine can be asymptomatic, GBS bacteriuria in certain populations can lead to UTI, pyelonephritis, and systemic infection. For example, in the elderly, where the incidence of invasive GBS has increased two- to fourfold in the past 20 years, 39% of GBS bacteremia cases are associated with concurrent GBS bacteriuria (Edwards and Baker, 2005). In pregnancy, GBS bacteriuria increases risk for intrapartum fever, chorioamnionitis, preterm delivery, and vertical transmission of the pathogen to the newborn (Patras and Nizet, 2018). Despite these risks associated with GBS bacteriuria, the mechanisms that drive GBS colonization and pathogenesis in the urinary tract remain poorly understood. The virulence regulator CovR and capsule production are both important during systemic infection and are also crucial for the establishment of bladder colonization (Kline et al., 2011; Kulkarni et al., 2013). However, another well-characterized virulence factor, β-hemolysin/cytolysin, may be dispensable under certain conditions, suggesting that GBS pathogenesis in the urinary tract may be unique compared to other tissues (Kulkarni et al., 2013; Leclercq et al., 2016; Sullivan et al., 2017). Recently, there has been a paradigm shift in the historical understanding that urine is typically sterile. In particular, altered bladder microbiota are observed in lower urinary tract pathologies including symptomatic UTI and incontinence (Nienhouse et al., 2014; Pearce et al., 2014). These data underscore that while a single primary organism, such as GBS, may be the underlying cause of clinical UTI symptoms, infection occurs in a polymicrobial environment that can influence pathogenesis and host response. For example, concurrent exposure of GBS promotes persistent uropathogenic *E. coli* (UPEC) infection of the urinary tract via modulation of the immune response (Kline et al., 2012). However, significant gaps still exist in our understanding of how the presence or absence of specific microbial organisms during initial inoculation, colonization, and infection may influence the course of disease.

*Candida albicans* is an opportunistic fungal pathogen estimated to colonize the urogenital tracts of 30% of women (Achkar and Fries, 2010). While certain conditions can predispose a patient toward pathogenic candiduria, such as age, pregnancy, diabetes mellitus, and catheterization, the presence of *C. albicans* in the urine is usually asymptomatic (Kauffman, 2005). Yet, *C. albicans* directly interacts with a number of bacterial uropathogens such as *E. coli, K. pneumoniae*, and GBS (Centeno et al., 1983; Levison and Pitsakis, 1987; Pidwill et al., 2018). In mucosal environments, such as the mouth and vaginal tract, *C. albicans-*bacterial interaction influences bacterial colonization, susceptibility to antibiotics, biofilm formation, and host responses (Allison et al., 2016), suggesting candiduria may also influence the pathogenesis of bacterial UTI. Moreover, GBS and *C. albicans* are frequently co-isolated from the vaginal tract, and synergistic interaction between GBS and *C. albicans* has been described during vaginal epithelial adherence *in vitro* (Bayó et al., 2002; Altoparlak et al., 2004; Pidwill et al., 2018). In this study, we provide evidence that candiduria can influence GBS colonization of the bladder by enhancing GBS interaction with the bladder epithelium.

## Materials and Methods

### Fungal Strains

Strains and sources of *C. albicans* used in this study are as follows: wild-type (WT) SC5314 (American Type Culture Collection, ATCC MYA2876), reference strain DAY185 (Nobile et al., 2008), *als3*Δ*/*Δ-mutant CAYF178U (Nobile et al., 2006), and the *ALS3*-complemented strain CAQTP178U (Nobile et al., 2006). Strains were inoculated into yeast-peptone-extract (YPD) broth (Difco #242720) and passaged twice at 30°C shaking before use in an experiment. On the day of experiment, overnight cultures (14–16 h) were washed twice in sterile phosphate-buffered saline (PBS) before preparing the infectious dose. Cell numbers were determined by counting on a hemocytometer. For all experiments involving *C. albicans* CFU counts, samples (lysates or mouse organs) were plated on Sabouraud Dextrose Agar with 75 mg/L chloramphenicol (SDA; Difco #210950) and incubated for 24–36 h at 30°C. For *C. albicans* cells labeled with CellTracker Blue (Thermo Fisher), yeast cells were washed once in PBS, resuspended at a concentration of 2 × 10^7^ cells/mL, and incubated with 30 μM CellTracker Blue for 20 min at 30°C, shaking. After incubation, cells were washed twice in PBS, then resuspended in RPMI prior to infection of HTB-9 cells.

### Bacterial Strains

The human serotype III group B *Streptococcus* strain COH1 (ATCC #BAA-1176) was used for all experiments. The GFP-expressing COH1 strain has been previously described (Cutting et al., 2014). An overnight culture of GBS strain COH1 was diluted 1:10 in 3 mL Todd-Hewitt broth (THB, Hardy Diagnostics #7161D) and incubated at 37°C until mid-log phase (defined as an OD_600_ of 0.4, ~2 × 10^8^ cells/mL). Erythromycin (5 μg/mL) was added to maintain the plasmid when growing the GFP-expressing GBS. At log-phase, cultures were centrifuged at 3,000×*g* for 5 min and washed in PBS prior to preparing samples for infection. For all experiments involving GBS CFU counts, samples were plated on CHROMagar StrepB plates (CHROMagar; DRG International, #SB282) and incubated for 24 h at 37°C.

### Animal Care

All animal experiments were conducted under veterinary supervision and approved by the University of California, San Diego Institutional Animal Care and Use Committee (IACUC). Eight- to ten-week-old female C57Bl6/J mice were purchased from Jackson Laboratories and allowed to acclimatize for 48 h before experiments. Mice were allowed to eat and drink *ad libitum*. All efforts were made to minimize suffering of animals employed in this study.

### *In vivo* Urinary Tract Infection

GBS or *C. albicans* (1 × 10^7^ CFU) in 100 μL PBS was prepared for mono-infection experiments. For co-infection, GBS (1 × 10^7^ CFU) and *C. albicans* (1 × 10^7^ CFU) were prepared together in a total volume of 100 μl PBS. Mice were infected via transurethral inoculation, as described previously (Coady et al., 2018). Twenty-four hours after infection urine was collected and plated for *C. albicans* and GBS CFU enumeration. Mice were humanely euthanized via CO_2_ asphyxiation, and bladders and kidneys were removed and homogenized in PBS using a MagNa Lyser (Roche). Organ lysates were serially diluted and plated for CFU enumeration of *C. albicans* and GBS.

### Adherence Assay

The human bladder epithelial cell line 5637 (ATCC HTB-9) was grown in RPMI-1640 with glutamine and 10% heat-inactivated fetal bovine serum (FBS). Cells were seeded in 24-well plates and grown to confluence overnight (~1 × 10^5^ cells/well). On the day of infection, HTB-9 cells were washed in PBS and media replaced with 400 μL of fresh RPMI-1640 (without FBS). Next, GBS (1 × 10^6^ CFU), *C. albicans* (1 × 10^6^ CFU), or GBS (1 × 10^6^ CFU) with *C. albicans* (1 × 10^6^ CFU) were prepared in 100 μL RPMI-1640 and added to cells, resulting in a multiplicity of infection (MOI) of 10 for all organisms. Infected cells were centrifuged at 300 × g for 5 min, then incubated at 37°C with CO_2_ for 45 min. After incubation, cells were washed 6 × with PBS, incubated in 0.25% trypsin for 10 min, and lysed by vigorous pipetting in 0.025% Triton-X in PBS. Lysate was plated for CFU enumeration of *C. albicans* and GBS. For experiments involving fixed epithelial cells, HTB-9 cells were fixed for 15 min in 4% paraformaldehyde prior to infection as described above.

### Visualization of GBS and *C. albicans* in Murine Bladders

Mice were infected with 1 × 10^7^ CFU of GFP-expressing COH1 GBS and *C. albicans*. Mice were euthanized 2 h post infection (as early as logistically feasible) in order to yield the highest possible number of organisms to visualize. Bladders were harvested, stretched, and fixed in 10% neutral-buffered formalin for 24 h and washed in PBS. Bladders were blocked for 15 min in 1% bovine serum albumin (BSA) in PBS prior to staining with 10 μg/mL anti-*C. albicans* antibody (ThermoFisher, #PA1-27158) for 30 min. Bladders were washed in PBS and stained with 5 μg/mL Alexa Fluor 594 goat anti-rabbit IgG secondary antibody (ThermoFisher, #A-11012) for 30 min, before incubating with 2 μM Hoechst 33342 Solution (ThermoFisher, #62249) for 15 min. Bladders were visualized immediately after staining on a Zeiss AxioObserver D1 microscope.

### Visualization of GBS and *C. albicans* Adherence to Bladder Epithelium *in vitro*

The human bladder epithelial cell line HTB-9 was grown in RPMI-1640 with glutamine and 10% heat-inactivated FBS. Cells were seeded in 24-well plates that contained sterile 15 mm glass coverslips (Fisherbrand) and grown to confluence overnight (~1 × 10^5^ cells/well). On the day of infection, HTB-9 cells were washed in PBS and media replaced with 400 μL of fresh RPMI-1640 (without FBS). Next, HTB-9 cells were infected with GFP-expressing GBS strain COH1 (GFP-GBS), *C. albicans* pre-labeled with 2 μM of CellTracker Blue (CTB-Ca), or GFP-GBS with CTB-Ca. For all experiments, the MOI of GFP-GBS was 10 with the CTB-Ca MOI changed as indicated (MOI of 10 or 1). Cells were centrifuged at 300 × g for 5 min, then incubated at 37°C with CO_2_ for 45 min. After incubation, cells were washed 6×with PBS, fixed for 20 min in 1% paraformaldehyde, and mounted on glass slides with ProLong Diamond AntiFade Mountant (Thermo Fisher). Coverslips were imaged with a Leica SP8 Super Resolution Confocal microscope and attached GFP-GBS and CTB-Ca were quantified using ImageJ.

### Statistical Analysis

All *in vitro* experiments were performed in at least three independent replicates, except for the microscopy experiments, which were repeated twice. All *in vivo* experiments were performed using at least five mice per group and repeated in at least two independent replicates. Statistical analyses were conducted using GraphPad Prism, version 8.3.0 (GraphPad Software Inc., La Jolla, CA). Mean values from technical replicates were used for statistical analyses, with independent experiment values or biological replicates represented in graphs with medians with or without interquartile ranges as indicated in figure legends. Statistical tests performed include nonparametric Mann-Whitney or Kruskal-Wallis one-way analysis of variance (ANOVA) with Dunn's multiple-comparison.

## Results

### *C. albicans* Increases GBS Colonization of the Murine Bladder

**Figure 1:**
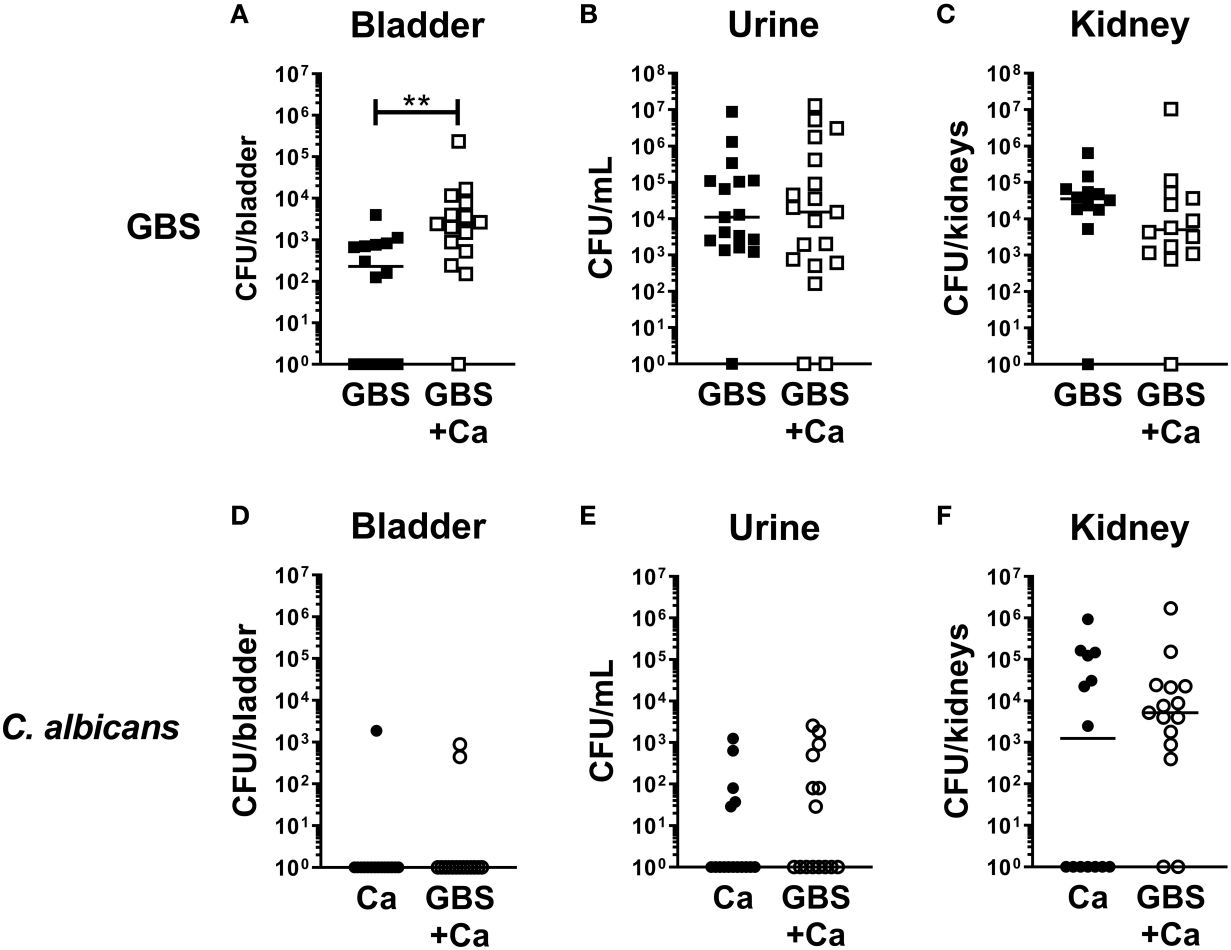
Presence of *C. albicans* during urinary tract infection increases GBS colonization of the bladder. Female C57Bl/6J mice were infected with 1 × 10^7^ CFU bacteria, fungi or a mixed culture of bacteria and fungi via transurethral inoculation. At 24 h, bladder, urine, and kidneys were harvested and assessed for bacterial **(A–C)** and fungal **(D–F)** growth. Squares represent individual mice, and lines represent the median for each group. Experiments were done three times independently and data combined (*n* = 14–15 mice per group). Statistical analysis between samples was performed using Mann-Whitney test. Significant comparisons are indicated; all others are not significant. ^**^P < 0.01.

To determine how *C. albicans* influences the pathogenesis of GBS UTI, we employed a murine model of polymicrobial infection where GBS and *C. albicans* are co-inoculated via transurethral injection. Female C57Bl/6J mice were infected with 1 × 10^7^ CFU GBS strain COH1, 1 × 10^7^ CFU WT *C. albicans* SC5314, or both organisms mixed together. Analysis of bacterial colonization in the bladders of mice at 24 h post-infection revealed that the GBS burden in mice co-infected with GBS and *C. albicans* was significantly higher (median: 2,400 CFU/bladder) than mice infected with GBS alone (median: 240 CFU/bladder, *P* = 0.0034) (Figure 1A). Interestingly, bacterial urine CFUs and colonization of the kidney were not enhanced by coinfection with *C. albicans* (Figures 1B,C). In contrast to bacterial colonization, coinfection had no measurable impact on *C. albicans* colonization of the bladder, urine, or kidney (Figures 1D–F). Collectively, these data suggest that the presence of *C. albicans* during GBS infection of the urinary tract promotes bacterial but not fungal colonization of the bladder.

### *C. albicans* Promotes GBS Adherence to Bladder Epithelium

**Figure 2:**
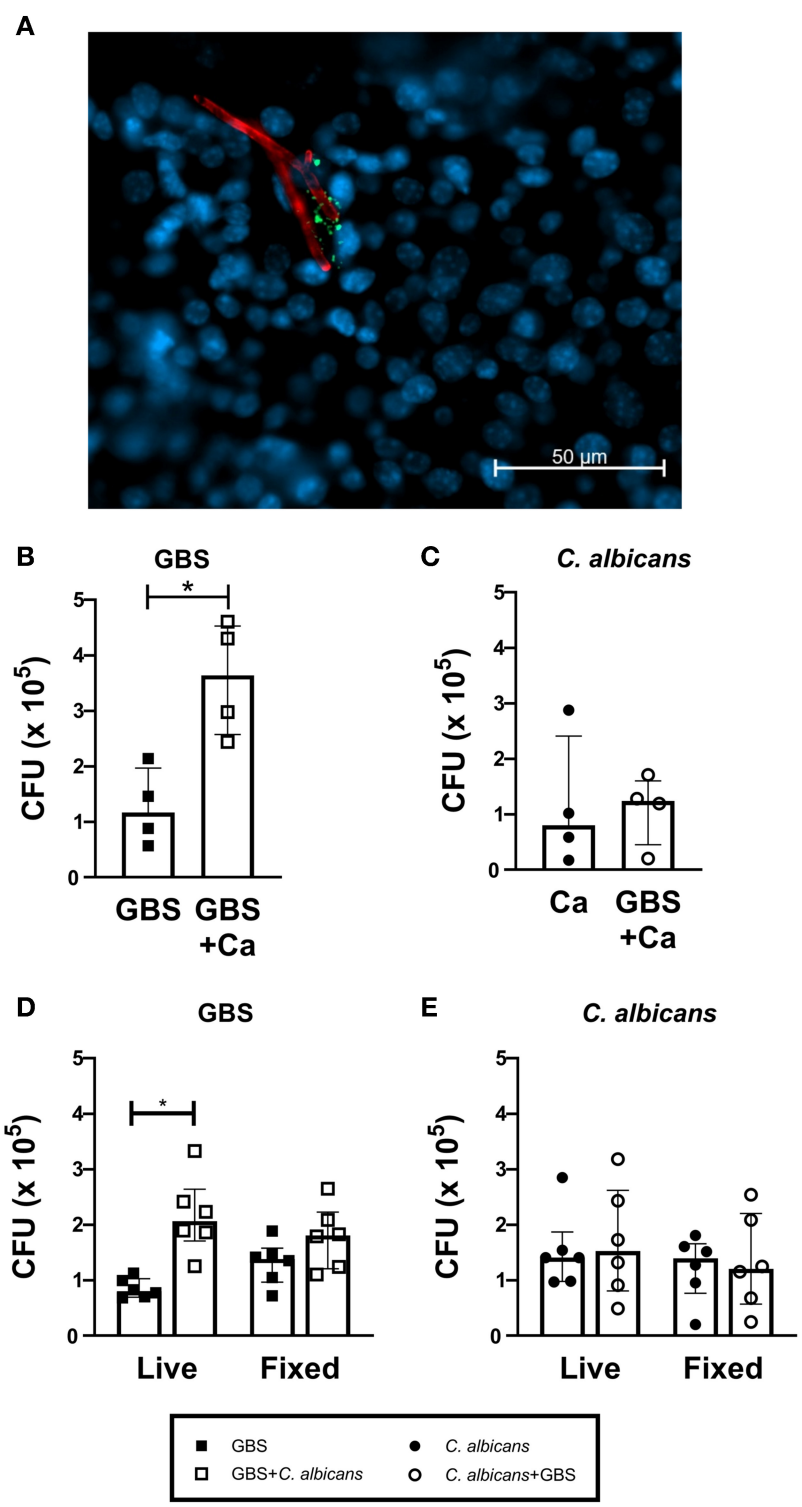
*C. albicans* and GBS coinfection increases bacterial adherence to bladder cells. **(A)** WT *C. albicans* SC5314 and GBS associate *in vivo*. C57Bl6/J female mice were infected with *C. albicans* and GFP-expressing GBS. Two hours later, bladders were harvested, fixed and stained for visualization via fluorescent microscopy. GFP-expressing GBS (green), AF594-anti-Ca antibody (red), and Hoechst dye (blue). Adherence of WT *C. albicans* SC5314 **(B)** and GBS **(C)** to human bladder epithelial cell line HTB-9 with single organism or mixed (GBS + *C. albicans)* inoculum. **(D)** GBS and **(E)** WT *C. albicans* SC5314 adherence of to live or fixed HTB-9 cells with single organism or mixed (GBS + *C. albicans*) inoculum. Data represent the means from four to five independent experiments performed in technical duplicate and are expressed as medians with interquartile ranges. Statistical analysis between experiments was performed using the Mann-Whitney test *P < 0.05.

To investigate if *C. albicans* and GBS interact in the bladder *in vivo*, we visually examined mouse bladders of mice co-infected with WT *C. albicans* SC5314 and GFP-expressing GBS (Cutting et al., 2014). We found instances where GBS was in close proximity to *C. albicans* hyphae (Figure 2A). To quantify if the presence of *C. albicans* can influence GBS interaction with the bladder epithelium, we utilized an *in vitro* infection model of the human bladder epithelial cell line HTB-9. When co-infected with WT *C. albicans* SC5314, GBS showed significantly increased adherence to HTB-9 cells (median: 3.6 × 10^5^ CFU) compared to GBS mono-infected cells (median: 1.2 × 10^5^ CFU, *P* = 0.0286) (Figure 2B). In accordance with our *in vivo* results, no increase in *C. albicans* adherence to bladder epithelium was observed with GBS co-infection (Figure 2C). To determine if increased GBS adherence depended upon a bladder epithelial response, adherence to fixed HTB-9 cells during coinfection was measured. Although these data demonstrated a moderate increase in GBS adherence to fixed cells upon coinfection with WT *C. albicans* SC5314 (Figure 2D, fixed cells, GBS alone median: 1.38 × 10^5^ CFU, GBS+WT *C. albicans* median: 1.81 × 10^5^ CFU, *P* = 0.0725), *C. albicans* promotion of GBS adherence was reduced compared to that seen with live HTB-9 cells (Figure 2D, live cells, GBS alone median: 8 × 10^4^ CFU, GBS + *C. albicans* median: 2.06 × 10^5^ CFU, *P* = 0.0007). This reduction in GBS adherence between coinfected live and fixed cells was not significant (Live GBS + *C. albicans* median: 2.06 × 10^5^ CFU, fixed GBS + *C. albicans* median: 1.81 × 10^5^, *P* = 0.1464), nor did *C. albicans* adherence change under any condition tested (Figure 2E). Collectively, our data suggest that the presence of *C. albicans* promotes bacterial colonization by increasing GBS adherence to bladder epithelium in a manner that is largely dependent on the host response.

### The *C. albicans* Adhesin Protein Als3 Mediates Increased GBS Adherence to Bladder Epithelium

**Figure 3:**
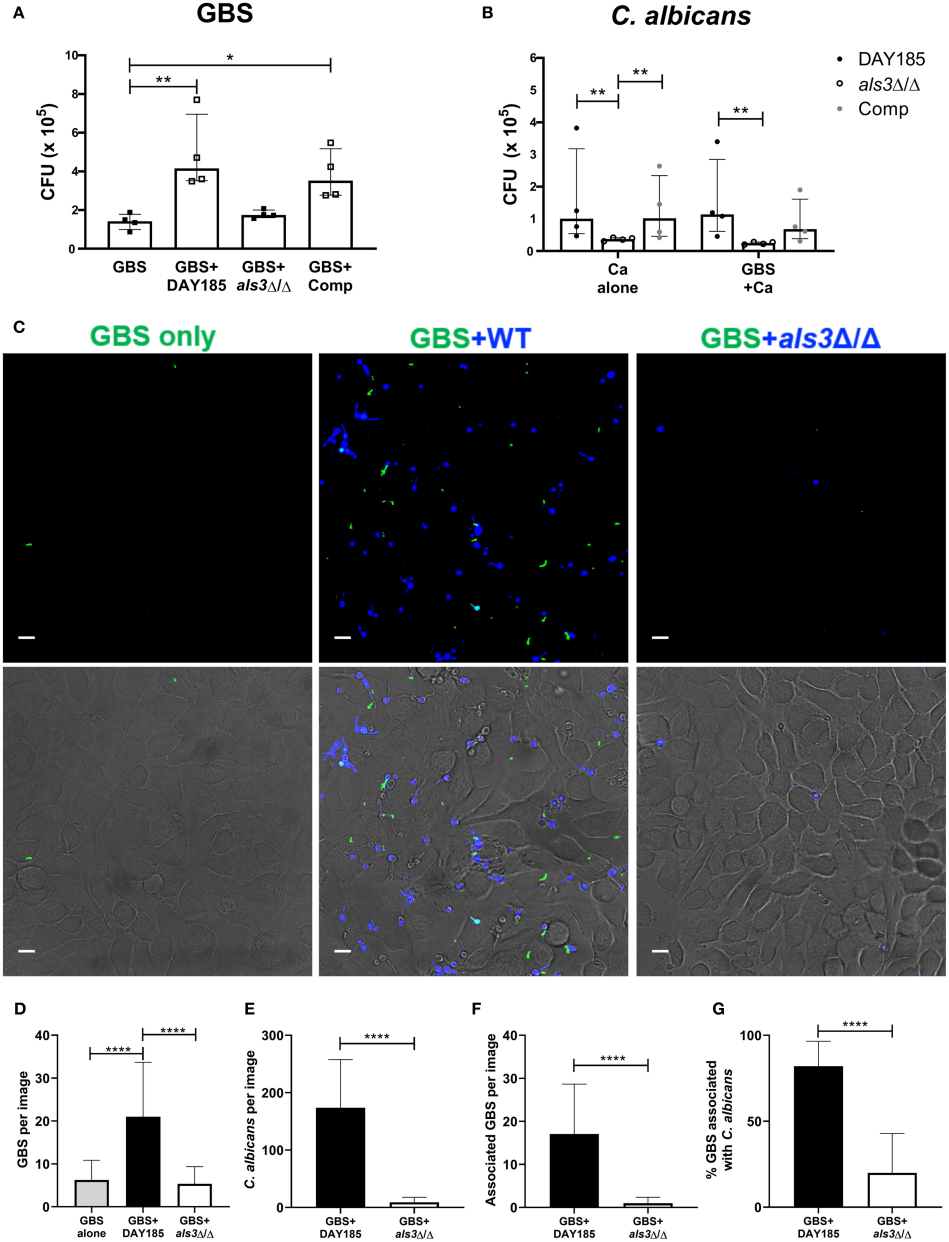
The fungal adhesin Als3 mediates adherence to bladder epithelium. **(A)** GBS recovery from HTB-9 bladder cells when infected alone or with wild-type (DAY185), *als3*Δ/Δ mutant, or *ALS3*-complemented (Comp) *C. albicans*. **(B)** Wild-type (DAY185), *als3*Δ/Δ mutant or *ALS3*-complemented (comp) *C. albicans* adherence to HTB-9 cells when infected alone or with GBS. Data represent the means from four independent experiments performed in technical duplicate and are expressed as medians with interquartile ranges. **(C–G)** HTB-9 cells were infected with GFP-expressing GBS only (green) or with GBS and CellTracker Blue-labeled *C. albicans* strains (blue). **(C)** Representative binding of GBS and *C. albicans* to HTB-9 cells. Merged images of green and blue fluorescence are shown in the top panel and merged fluorescence and phase images are shown in the bottom panel, scale bar = 10 μm. Data represents the quantification of **(D)** average GBS, **(E)** *C. albicans*, and **(F)** *Candida*-associated GBS in 15 fields/sample. **(G)** Percentage of total GBS that associates with indicated *C. albicans* strain. Data represents the combined image counts of two experiments performed in technical duplicate and are expressed as the mean with standard deviation. Statistical analysis was performed using the Kruskal-Wallis ANOVA with Dunn's multiple comparison or Mann-Whitney test, *P < 0.05, **P < 0.01, ****P < 0.0001.

In vaginal colonization, the fungal adhesin Als3 has been shown to mediate *C. albicans*:GBS interaction and *C. albicans* adherence to the epithelium (Pidwill et al., 2018). To assess if this adhesin also facilitates *C. albicans*:GBS interaction during bladder colonization, we compared the ability of an Als3-deficient *C. albicans* strain (*als3*Δ/Δ) and a WT *C. albicans* reference strain DAY185 to promote GBS adherence to bladder epithelium in our HTB-9 *in vitro* co-infection model. Consistent with our findings using WT *C. albicans* SC5314, GBS adherence was significantly enhanced in the presence of DAY185 (GBS alone median: 1.4 × 10^5^ CFU, GBS + DAY185 median: 4.2 × 10^5^ CFU, *P* = 0.0089, Figure 3A), while GBS adherence was not impacted when co-cultured with the *als3*Δ/Δ mutant (median: 1.7 × 10^5^ CFU, *P* > 0.9999). Additionally, *ALS3-*complementation of the *als3*Δ/Δ mutant restored the WT phenotype (median: 3.5 × 10^5^ CFU, *P* = 0.0347, Figure 3A). The *als3*Δ/Δ mutant showed decreased adherence (*als3*Δ/Δ alone median: 3.75 × 10^4^ CFU) to HTB-9 cells compared to WT *C. albicans* DAY185 (Figure 3B, DAY185 median: 1.01 × 10^5^ CFU, *P* = 0.0029). Coinfection with GBS did not alter adherence of either WT *C. albicans* DAY185 or the *als3*Δ/Δ mutant to HTB-9 cells (Figure 3B). To examine whether the difference observed in GBS adherence was facilitated by GBS binding to *C. albicans* or directly to the bladder epithelial cells, we visualized and quantified the amount of GBS adhered to HTB-9 cells directly, as well as the amount of adhered GBS associated with WT *C. albicans* DAY185 or the *als3*Δ/Δ mutant. We found a greater number of GBS adhering to bladder cells when coinfected with WT *C. albicans* DAY185 than with the *als3*Δ/Δ mutant (Figures 3C–G). In line with a role for Als3 in promoting *Candida*:GBS interactions, the percent of adherent GBS associated with *C. albicans* in *als3*Δ/Δ mutant was also significantly reduced compared to WT DAY185 (Figure 3G). As the *als3*Δ/Δ mutant is known to display decreased fungal adherence to bladder epithelium, we also repeated these experiments with a lower MOI of WT DAY185 (MOI = 1) to achieve a similar adherence to the *als3*Δ/Δ mutant (MOI = 10) (Supplemental Figure 1A). In this setting, we found that even at similar levels of *C. albicans* colonization, WT DAY185 (MOI = 1) had significantly more total GBS and *Candida*-associated GBS adherence than the *als3*Δ/Δ mutant (MOI = 10) coinfection (Supplemental Figures 1B,C). In addition, even when accounting for differences in fungal adherence by lowering the MOI, the percent of adherent GBS associated with *C. albicans* in WT DAY185 (MOI = 1) coinfection was higher than that with *als3*Δ/Δ mutant (MOI = 10) (Supplemental Figure 1D). Our data suggest that Als3 contributes to GBS:*C. albicans* association during infection of bladder epithelium and is important in increasing *C. albicans*-dependent GBS adherence to bladder epithelium.

**Figure 4:**
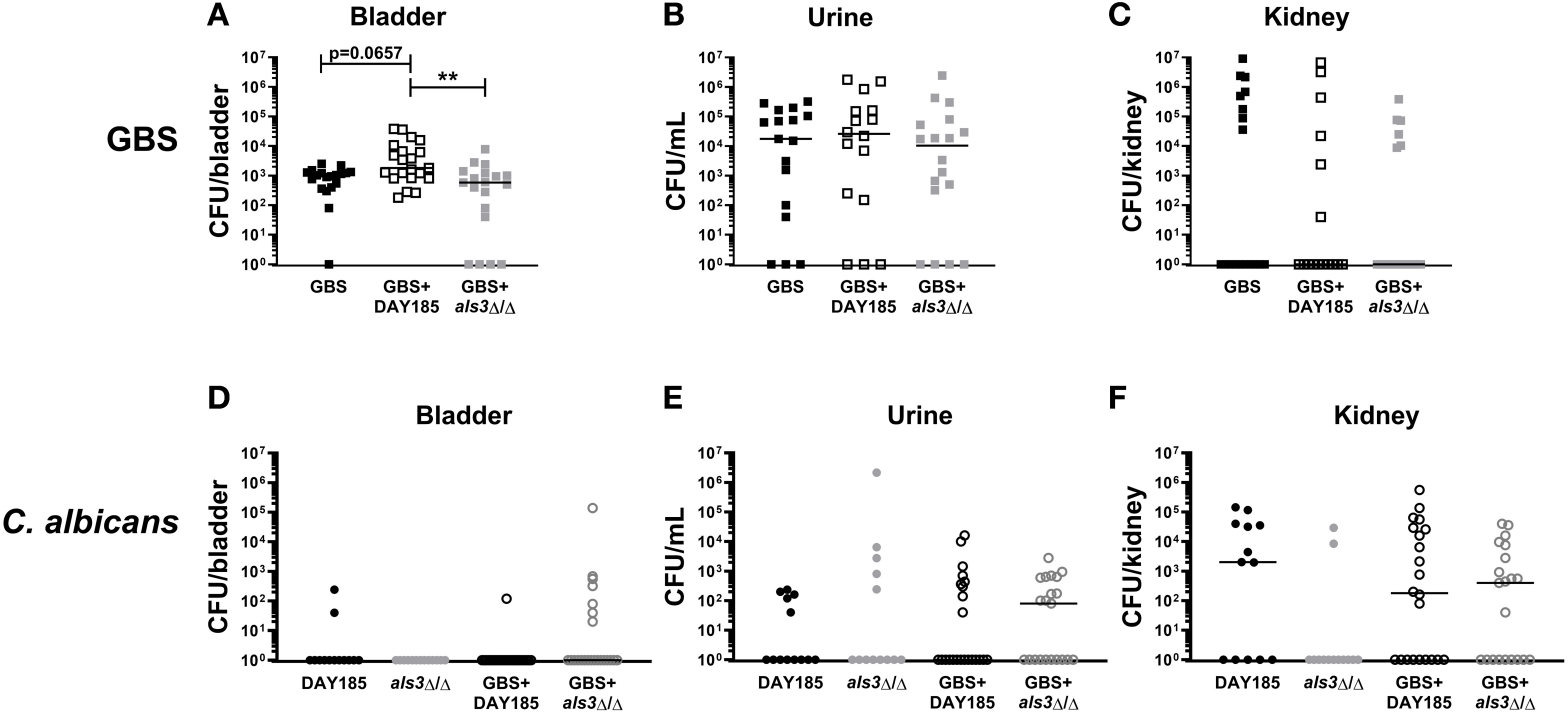
Loss of Als3 decreases bacterial colonization of bladder during *in vivo* coinfection. Mice were infected with GBS alone, *C. albicans* DAY185 + GBS, or *C. albicans als3*Δ/Δ + GBS. At 24 h, bladder, urine and kidneys were harvested and plated for GBS CFUs **(A–C)** and *C. albicans* CFU **(D–F)**. Infections were performed three times independently and data combined (*n* = 16–18 mice per group). Statistical analysis between experiments was performed using the Kruskal-Wallis ANOVA with Dunn's multiple comparison **P < 0.01.

To validate the importance of Als3 for *in vivo* colonization, we performed transurethral co-inoculation of GBS with DAY185 or *als3*Δ/Δ *C. albicans*. Although not statistically significant, mice inoculated with WT *C. albicans* DAY185 and GBS display a trend toward increased GBS colonization (Figure 4A, GBS + DAY185 median: 1800 CFU) compared to mice infected with GBS alone (GBS alone median: 1030 CFU, *P* = 0.0657). *als3*Δ/Δ *C. albicans* failed to promote GBS bladder colonization (Figure 4A, GBS + *als3*Δ/Δ median: 590, *P* = 0.770). No difference in bacterial colonization was observed in the urine or kidneys of coinfected mice compared to mice infected with GBS alone (Figures 4B,C). Levels of *C. albicans* colonization of the bladder, kidneys, and urine for both WT DAY185 and the *als3*Δ/Δ mutant were similar to colonization levels observed for WT *C. albicans* SC5314 (Figures 4D–F). Collectively, the work outlined in this manuscript demonstrates that the presence of *C. albicans* during GBS UTI promotes bacterial colonization of the bladder by increasing GBS adherence to the bladder epithelium.

## Discussion

Historically, UTI have been studied and treated as monomicrobial; however, this may not accurately reflect the diverse microbial communities present in the urinary tract and in microbial reservoirs, such as the gastrointestinal and vaginal tracts. Here, using both *in vitro* and *in vivo* models of coinfection, we show that bacterial co-inoculation with *C. albicans* promotes GBS bladder colonization through increased adherence to the bladder epithelium. Our work suggests increased adherence involves the *C. albicans* adhesin Als3, a protein previously described to directly promote GBS:*C. albicans* interaction (Pidwill et al., 2018). Importantly, this work expands our understanding of how the presence of distinct microbial organisms can influence bacterial colonization and UTI pathogenesis.

Adherence is a critical first step in microbial colonization of the host for both pathogenic and commensal organisms. Interaction between microbial species is one of numerous identified strategies used by both bacteria and fungi to increase adherence to host substrates. For example, *C. albicans* can associate with multiple *Streptococcus* and *Staphylococcus* species, aiding in their adherence and biofilm-formation (Lohse et al., 2017; Koo et al., 2018). For some streptococcal species, like *S. gordonii*, bacterial surface adhesins bind directly to *Ca*-Als3 to mediate bacterial-fungal interaction (Silverman et al., 2010). GBS also interacts with *C. albicans* through recognition of adhesins. In particular, Als3 can bind GBS Bsp proteins, and this interaction is critical for the ability of *C. albicans* to promote GBS binding to vaginal epithelium (Pidwill et al., 2018). Although multiple Bsp proteins have been described in invasive isolates of GBS (Rego et al., 2016), the presence and diversity of Bsp proteins in urinary isolates of GBS have not been described. Future studies will be necessary to discern if GBS adherence to *C. albicans* and the epithelium during bladder infection is mediated through specific Bsp proteins.

In this study, we confirmed that Als3 expression is critical for *C. albicans* binding to bladder epithelium (Coady et al., 2018). Our data also support a role for *Ca*-Als3 in facilitating GBS adhesion to bladder epithelium. While we observed an increase in GBS associated with WT *Candida* during infection of bladder epithelial cells (Figure 3), we also found that the *C. albicans*-induced increase in GBS adhesion to bladder epithelial cells was diminished when the cells were fixed (Figure 2). This result suggests that *C. albicans* promotes GBS adherence through a manner dependent on both *Candida* Als3 expression and induction of a host response in the bladder epithelium. However, the extent to which these two phenomena are linked remains uncertain, and the nature of the host response and whether it depends on Als3-driven interactions between GBS and *Candida* are outstanding questions. Both *in vitro* and *in vivo* interaction of *C. albicans* with epithelial cells induces invasion, cellular damage, and a pro-inflammatory response, largely through the action of the cytolytic peptide toxin candidalysin (Moyes et al., 2016; Naglik et al., 2017). Likewise, GBS deficient in either production or regulation of β-hemolysin/cytolysin are less able to colonize the urogenital tract (Kulkarni et al., 2013; Patras et al., 2013; Leclercq et al., 2016; Sullivan et al., 2017). These data suggest that pathogen control of host cell damage and inflammation are important factors in determining bladder persistence. The mechanisms that influence *C. albicans*-dependent changes in the host environment to promote GBS colonization remain a research area of interest.

In contrast to adherence to the vaginal epithelium, the GBS:*C. albicans* interaction did not appear to promote *C. albicans* adhesion to bladder cells or increase *C. albicans* colonization of the bladder, indicating that the interaction is not mutually synergistic. This may be an accurate reflection of what occurs in humans, since *C. albicans* is a common commensal of the gastrointestinal and vaginal tract but rarely observed in the urine of healthy humans. While *C. albicans* may be introduced into the urinary tract with GBS, it likely results in transient colonization. Populations at risk for infection by *C. albicans* and GBS include pregnant women, the elderly, diabetes mellitus patients, and catheterized patients (Edwards and Baker, 2005; Achkar and Fries, 2010), but in these susceptible patients, candiduria is often considered benign and non-significant, and thus may be under-reported (Kauffman, 2005). As such, although *C. albicans* has not been clinically implicated in complications involving GBS, our work suggests that the urogenital carriage of *Candida* can promote the colonization of GBS, and thus may increase disease incidence and/or severity in these patients. Understanding the associative roles of *C. albicans* and other colonizing microbes in urine may reveal additional complementary interactions that play a role in establishing UTI.

## Data Availability Statement

The datasets generated for this study are available on request to the corresponding author.

## Ethics Statement

The animal study was reviewed and approved by the University of California, San Diego Institutional Animal Care and Use Committee.

## Author Contributions

SS and AC planned and performed experiments, analyzed data, and wrote the manuscript. ARR, AMR, SM, and KP performed experiments. KP, AMR, and VN provided input into experimental planning and edited the manuscript.

### Conflict of Interest

The authors declare that the research was conducted in the absence of any commercial or financial relationships that could be construed as a potential conflict of interest.
