# Supplementary material for: The Fungal Pathogen Candida albicans Promotes Bladder Colonization of Group B Streptococcus
Source: Front Cell Infect Microbiol. 2020 Jan 10;9:437. doi: 10.3389/fcimb.2019.00437 (PMC6966239; doi:10.3389/fcimb.2019.00437)
Supplement: Supplementary file 1 [file Data_Sheet_1.pdf]

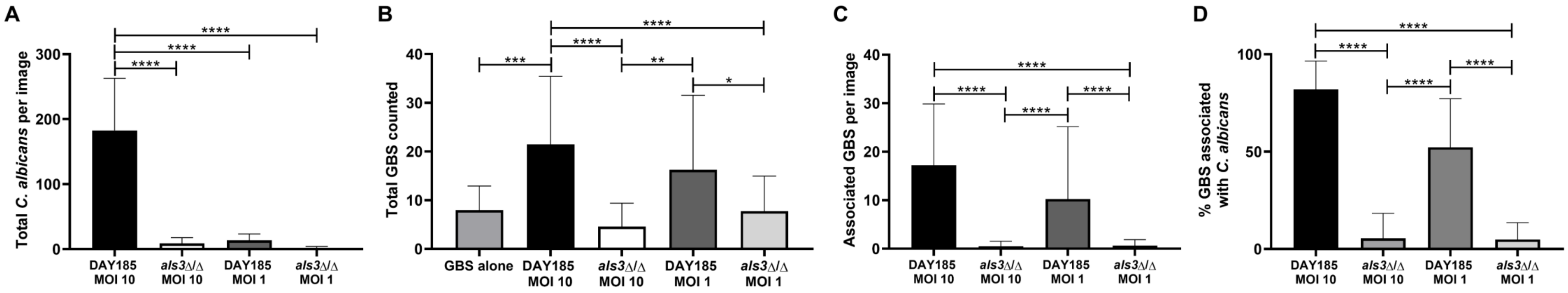

Supplemental Figure 1. **The fungal adhesin Als3 mediates Ca:GBS interaction to bladder epithelium.** HTB-9 cells were infected with GFP-expressing GBS, or the same number of GBS together with coinfection of fluorescently labeled *C. albicans* either at a MOI of 1 or 10. Cells were imaged with a fluorescence microscope and total GBS **(A)**, *C. albicans* **(B)**, and *Candida*-associated GBS **(C)** quantified. **(D)** Bars represent the % of total GBS that visually associated with the indicated *C. albicans* strain. Data represent the average image counts of one experiment performed in technical duplicate, with 30 images analyzed per condition. * $p<0.05$, **$p<0.01$, **$p<0.001$, ***$p<0.005$, ****$p<0.0001$
